# Cog-Wheel Octameric Structure of RS1, the Discoidin Domain Containing Retinal Protein Associated with X-Linked Retinoschisis

**DOI:** 10.1371/journal.pone.0147653

**Published:** 2016-01-26

**Authors:** Martin Bush, Dheva Setiaputra, Calvin K. Yip, Robert S. Molday

**Affiliations:** Department of Biochemistry and Molecular Biology, University of British Columbia, Vancouver, British Columbia, Canada; University of Cologne, GERMANY

## Abstract

RS1, also known as retinoschisin, is a disulphide-linked, discoidin domain containing homo-oligomeric protein that plays a crucial role in maintaining the cellular and synaptic organization of the retina. This is highlighted by the finding that over 130 mutations in RS1 cause X-linked retinoschisis, a retinal degenerative disease characterized by the splitting of the retinal cell layers, disruption of the photoreceptor–bipolar synapses, degeneration of photoreceptors, and severe loss in central vision. In this study, we investigated the arrangement of the RS1 subunits within the oligomer complex using single particle electron microscopy. RS1 was seen as two stacked rings with each ring displaying a symmetrical cog wheel-like structure with eight teeth or projections corresponding to the RS1 subunits. Three dimensional reconstruction and molecular modelling indicated that the discoidin domain, the principal functional unit of RS1, projects outward, and the Rs1 domain and C-terminal segment containing intermolecular disulphide bonds are present in the inner ring to form the core octameric structure. These studies provide a basis for further understanding the role of the novel core RS1 octameric complex in retinal cell biology and X-linked retinoschisis.

## Introduction

The vertebrate retina is a light-sensitive tissue at the back of the eye that functions in the initial steps of vision. It is composed of three neural cell layers (photoreceptors, inner retinal neurons, ganglion cells) interconnected by two synaptic layers (outer and inner plexiform layers). Retinal glial cells known as Mueller cells span the width of the retina providing mechanical and environmental support for the retinal neurons. This cellular organization is essential for the efficient conversion of light into electrical signals in rod and cone photoreceptors and the processing of these signals by secondary retinal neurons for transmission to the brain for image perception.

The molecular basis for generating and maintaining the unique cellular architecture of the retina is not well understood. However, analysis of the retina in its normal and disease states indicates that the extracellular protein RS1 also known as retinoschisin plays a crucial role in maintaining the cellular and synaptic organization of the retina. Mice deficient in RS1 display cystic cavities in the inner retina, disruption of the photoreceptor-bipolar synapse, progressive photoreceptor degeneration, and a reduction in the b-wave amplitude of the electroretinogram (ERG) indicative of decreased signal transmission from photoreceptors to bipolar cells [1–4]. Individuals with X-linked retinoschisis (XLRS), an early onset macular degeneration associated with mutations in RS1, show similar features including the splitting of the retinal layers most pronounced in the macula, a reduction in the ERG b-wave amplitude, and significant loss in central vision [5, 6]. Over 130 mutations have been linked to XLRS with the majority being missense mutations [7, 8].

**Fig 1:**
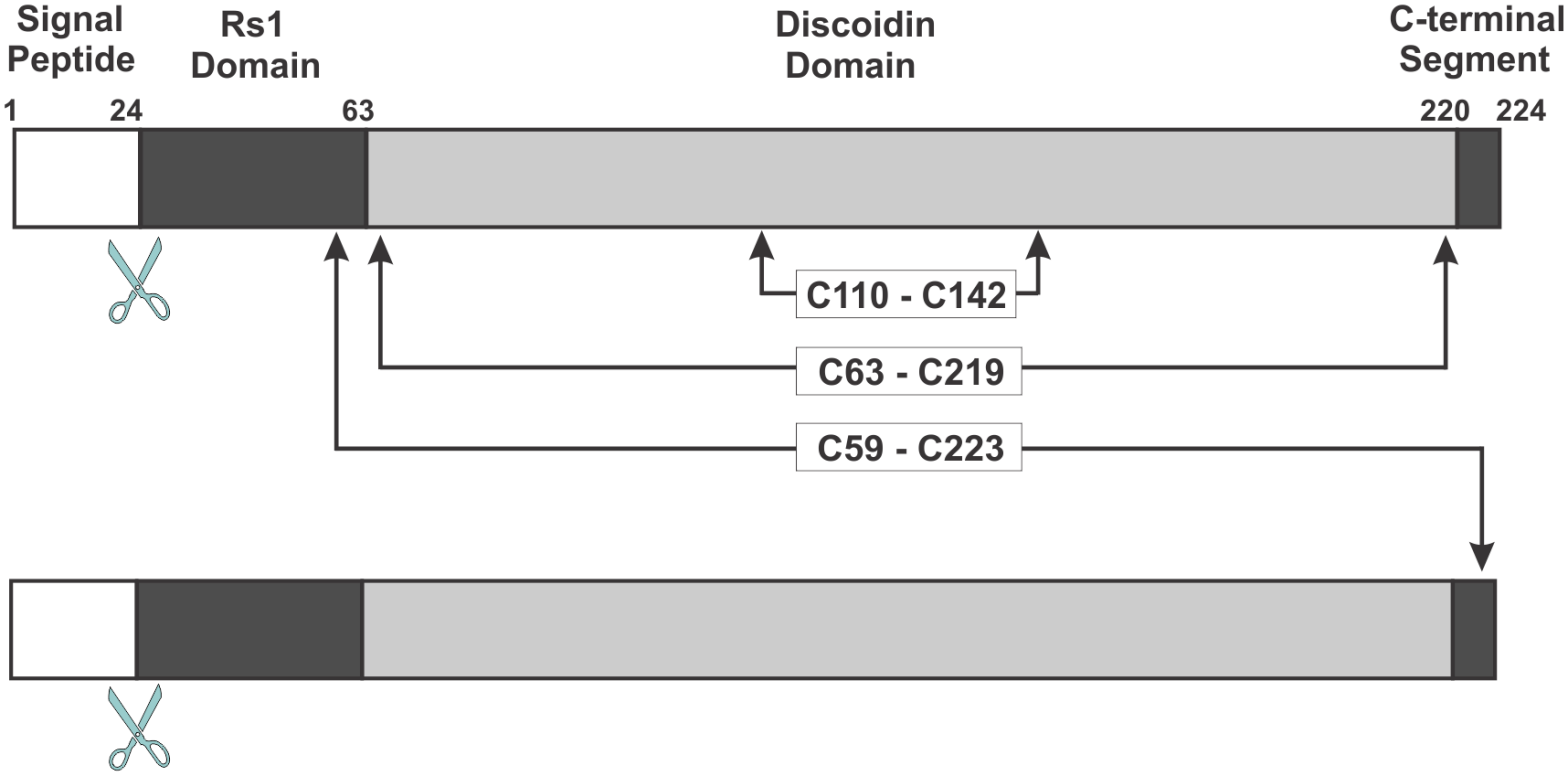
Linear diagram of two RS1 subunits showing the various domains and key disulphide bonds. The signal peptide (1–23 amino acids) is cleaved during biosynthesis. In the mature protein there are two intramolecular disulphide bonds (C63 –C219; C110 –C142) and one intermolecular disulphide bond (C59 –C223) responsible for homo-oligomeric assembly of subunits.

The *RS1* gene encodes a 224 amino acid polypeptide consisting of a 23 amino acid N-terminal signal sequence and a 157 amino acid discoidin domain flanked by an upstream 39 amino acid Rs1 domain and a downstream 5 amino acid C-terminal segment (Fig 1) [8, 9]. During protein synthesis, the signal sequence is cleaved to produce a 201 amino acid polypeptide chain. The mature polypeptide is secreted from photoreceptors and bipolar cells as a homo-oligomer held together by intermolecular disulphide bonds [10]. Intermediate complexes present in RS1 mutant proteins have led to the view that RS1 exists as a homo-octameric complex [11].

Although biochemical studies together with molecular modelling of the discoidin domain of RS1 have provided insight into some of the structural features of RS1 [10–13], the octameric complex has not been confirmed through independent methods and the arrangement of subunits within the complex has not been investigated. In this study we have used single particle electron microscopy together with molecular modelling to gain insight into the structure of the RS1 complex. We show that the core RS1 structure is composed of eight subunits arranged in a cog-wheel or sprocket-like arrangement. Molecular modelling of the RS1 subunit provides insight into the orientation and arrangement of RS1 subunits within this structure.

## Materials and Methods

### Expression and Purification of RS1

Human RS1 was expressed and secreted from stably transformed Sf21 insect cells grown in culture for 96 hours [14]. Anion exchange and galactose affinity chromatography were used to purify RS1 from 1 litre of ESF-AF culture media (Expression Systems) as previously described [14]. An additional size exclusion chromatography (SEC) step was introduced after the galactose affinity chromatography as follows. Protein eluted from the galactose agarose column in 1M IPTG, 100mM sodium chloride and 20mM Tris-HCl, pH 7.5, was concentrated to 500μl, loaded on to a Superdex 200 10/300 column (GE Lifesciences), and run at 0.5ml/min flow rate controlled with an AKTA Pure FPLC. The column was buffered with 20mM Tris-HCl, pH 7.5, and 100mM NaCl. After pooling the peak fractions, the protein was concentrated to 0.1mg/ml using centrifugal filters with a mass cut-off of 10,000 kDa. Protein standards (GE lifesciences HMW kit) were run on the same column in the same buffer for molecular mass calibration.

### Negative stain electron microscopy

Negatively stained specimens were prepared from the purified RS1 as previously described [15]. In brief, 3 μl of the purified protein was adsorbed to glow discharged carbon-coated copper grids, stained with 0.75% (w/v) uranyl formate solution, and the specimens were air dried. These specimens were examined using a Tecnai Spirit transmission electron microscope (FEI) equipped with a LaB6 filament and operated at an accelerating voltage of 120 kV. Micrographs were obtained at a nominal magnification of 49,000X with an FEI Eagle 4K x 4K charge-coupled device (CCD) camera at a defocus value of -1.2 μm using low-dose procedures.

### Image processing

2 x 2 pixels from the electron micrographs were averaged for a final pixel size of 4.7Å at the specimen level. Single particle images were extracted from the micrographs using Boxer [16]. For two dimensional (2D) analysis, the selected particles (10,664 total) were subjected to reference-free alignment and K-means classification using the SPIDER image processing suite [17]. Fifty classes were specified (S1 Fig).

To produce the RS1 3D reconstruction, class averages yielding intact rings were used to generate initial 3D models using EMAN2 [18]. The initial models were then refined with images containing a single well-defined particle each (2,200/10,664 total particles) with RELION while enforcing D8 symmetry [19]. The final resolution was determined in RELION using the Fourier Shell Correlation (FSC) function using the 0.143 FSC criterion.

### Homology modelling

A model of the RS1 protein was built using the Phyre2 Protein fold recognition server (http://www.sbg.bio.ic.ac.uk/phyre2/html[20]). The search input was the mature peptide lacking the 23 amino acid leader sequence i.e. residues 24–224 in the intense mode. Further refinement and adjustment was performed using Coot software [21] to make the appropriate disulphide bonds within the molecule.

## Results

### Improved purification protocol

**Fig 2:**
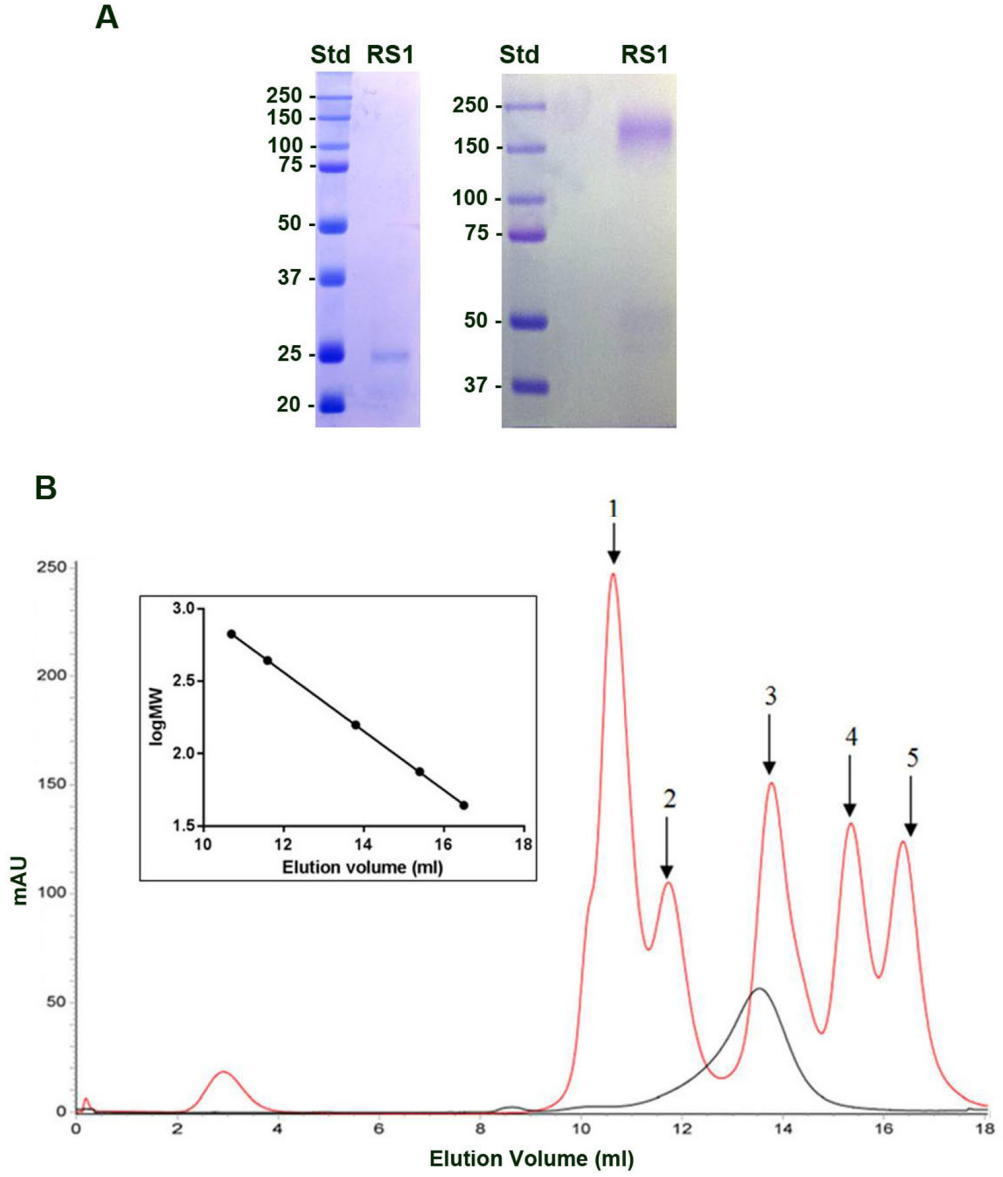
Analysis of RS1 by SDS gel electrophoresis and size exclusion chromatography (SEC). RS1 was purified from the culture fluid of Sf21 insect cells by ion exchange chromatography followed by galactose affinity chromatography and SEC. A. The sample was analysed on SDS gels under disulphide reducing (left) and nonreducing (right) conditions. B. Nondenatured RS1 sample was subjected to size exclusion chromatography on a calibrated column. Standards were 1: Thyroglobulin 669KDa, 2: Ferritin 440kDa, 3: Aldolase, 158kDa, 4: Conalbumin 75kDa, 5: Ovalbumin 44kDa. Inset: Plot of the Log MW vs. elution volume. RS1 (black trace) eluted with an apparent molecular mass of 180 kDa.

In initial studies, recombinant RS1 secreted from Sf21 insect cells was purified by ion exchange and galactose affinity chromatography. We attempted to crystalize the purified RS1 but were unsuccessful after extensive screening of different conditions. We subsequently analysed recombinant RS1 by single particle electron microscopy. The sample showed a few cog-wheel-like structures along with nondescript particles and disordered aggregates. The purity of RS1 was improved by introducing an additional size exclusion chromatographic (SEC) step following the galactose affinity chromatographic step. RS1 eluted from the SEC column as a single major peak. The purity of the sample in the pooled fractions was confirmed by SDS gel electrophoresis (Fig 2A). RS1 migrated as a band with an apparent molecular mass of about 25 kDa under disulphide reducing conditions and about 200 kDa under non-reducing conditions. The difference in size under reducing and nonreducing conditions supports the existence of disulphide linked oligomer structure as previously reported [11]. The size of RS1 was also determined under nondenaturing conditions on a calibrated SEC column (Fig 2B). RS1 eluted as a protein having an apparent molecular mass of ~180 kDa.

### Octameric Structure of RS1

**Fig 3:**
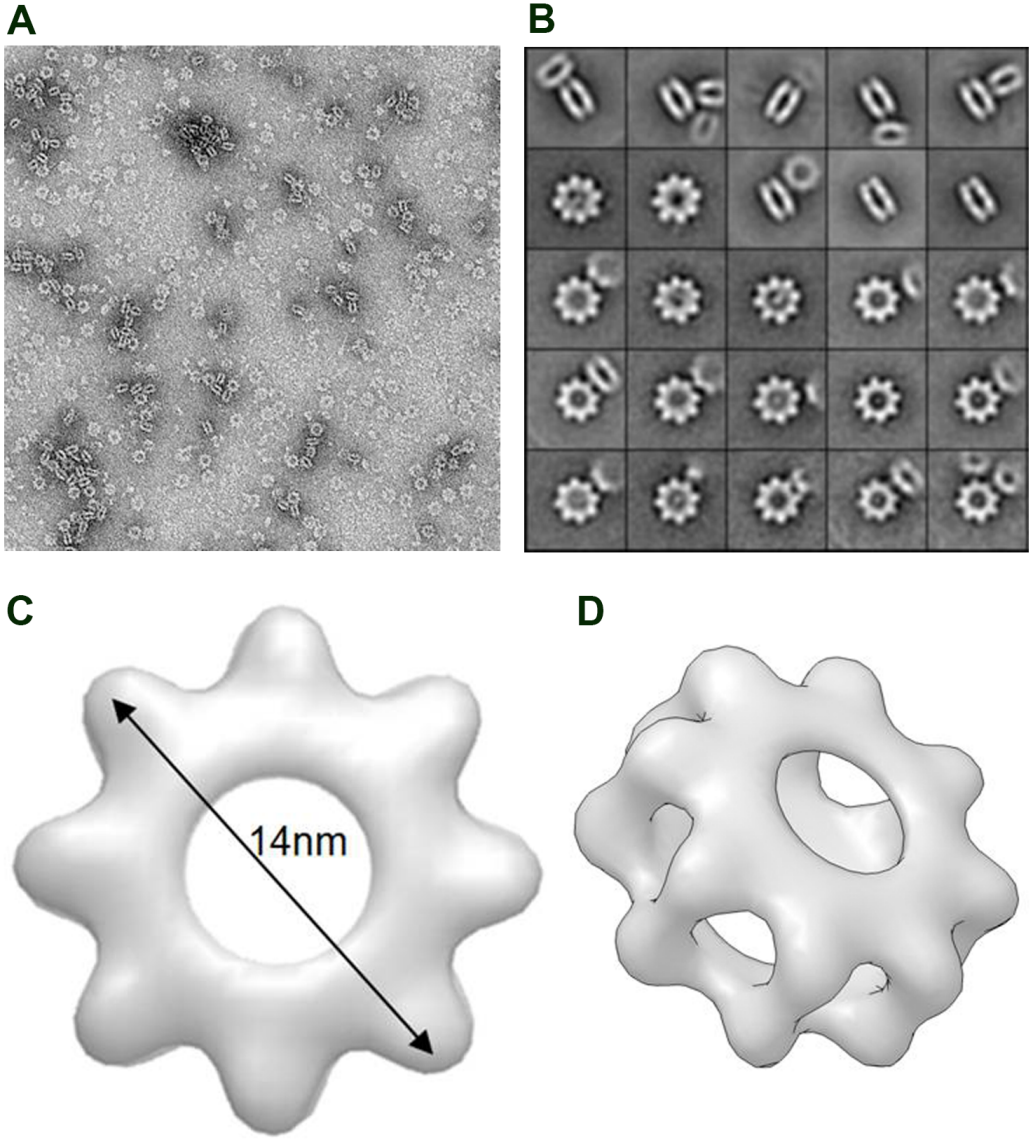
Single particle EM images and 3-D reconstruction of RS1. A. Electron micrograph of purified RS1. B. Represented refined images of RS1 in 2-dimensions. On-face images show a cog-wheel-like structure. Each box width = 30 nm. Also present are side views of double cog-wheel-like structures. C. 3-D on-face reconstruction from 10,664 EM images. D. Tilted 3-D image of a double cog-wheel showing interaction between two cog-wheels at the outer edge.

The raw electron micrographs of negative stained RS1 revealed particles with an apparent symmetrical cog-wheel or sprocket-like structure with eight discrete densities protruding from the ring (Fig 3A and 3B). Subsequent 2D classification and averaging analysis confirmed this observation and further showed that the oligomeric RS1 has an outer diameter of 14nm with a central cavity of 5nm. We also noticed some side views representing an on-edge presumably double cog-wheel structure with a thickness of 8nm. It is not possible to conclude whether all of the top down views represent double-ringed structures.

We next produced three dimensional reconstructions by generating the initial models using the “top down” and side views by EMAN and then iteratively refining the structure with particle images from the full dataset using Relion (Fig 3C and 3D). The resulting reconstruction showed a stacked cog-wheel architecture with the two rings interacting primarily at the outer ends of the cogs (Fig 3D).

### Homology modelling

**Fig 4:**
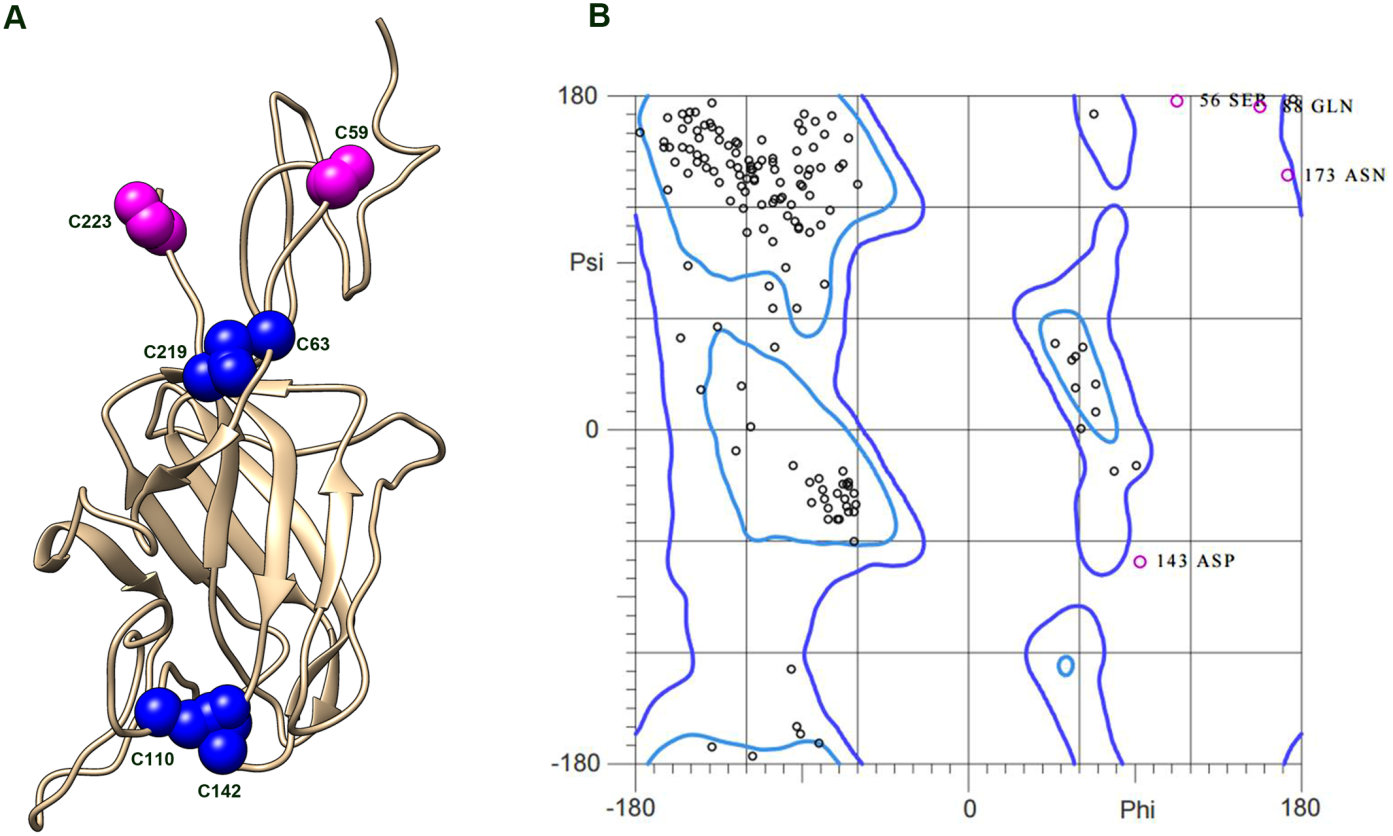
Molecular model of the mature RS1 subunit. A. The RS1 subunit model shows the cysteine residues involved in intramolecular disulphide bonds (C110-C142 and C63-C219) in blue and two cysteine residues involved in intermolecular disulphide bonds (C59 and C223) that generate the octamer complex in magenta. B. Ramachandran plot for the RS1 model.

To determine how the RS1 subunits are arranged into the cog-wheel structure, we first constructed a molecular model of the RS1 subunit using the bovine factor V C2 domain as a reference since residues 1839–2016 (PBD: 1sdd chain B) showed 31% sequence identity with residues 37–224 of RS1. The core discoidin domain is seen as a distorted barrel with a 5-stranded antiparallel β-sheet packed against a 3-stranded antiparallel β-sheet (Fig 4A). Three spikes or loops, which form a cavity that serves as a binding site for its ligand phosphatidylserine in Factor V, are present at one end of the RS1 discoidin domain. At the opposite end short segments join the β-strands.

As the missing region (residues 24 to 37) encompasses a relatively short stretch of sequence, we were able to model this segment using intermolecular and intramolecular disulphide bonds identified in biochemical studies [10, 11] as constraints. We have previously observed that the C59 residue in the Rs1 domain forms an intermolecular disulphide bonds with C223 residue in the C-terminal segment of an adjacent subunits (Fig 1) [10, 11]. This observation indicated that these cysteine residues must be located at an accessible surface and most likely on opposite sides of RS1 to allow for intermolecular disulphide bond formation and assembly into an octamer. We also added intramolecular disulphide bridges C110-C142 and C63-C219 to the model [10]. A disulphide bond between cysteine residues at the start and end of the discoidin domain (C63-C219 in RS1) has been observed in Factor V and other discoidin domains for which a structure is known [22, 23]. Mutagenesis and mass spectrometry have confirmed the presence of an additional intramolecular disulphide bonds between C110 in spike 2 with C142 in spike 3 (Fig 4A)[10, 11]. The RS1 model was analysed using the MolProbity program [24] to assess the quality of the final structure. Over 85% of the residues are in favourable regions suggesting overall a properly refined stereochemistry for the molecule. All the outliers are residues found on the surface of the protein and appear to be in regions with little secondary structure (Fig 4B). In summary, the generated model shows that RS1 adopts an overall wedge shape with the discoidin domain occupying the wider end and the Rs1 domain present in the narrow end.

### Subunit arrangement within the RS1 octamer

**Fig 5:**
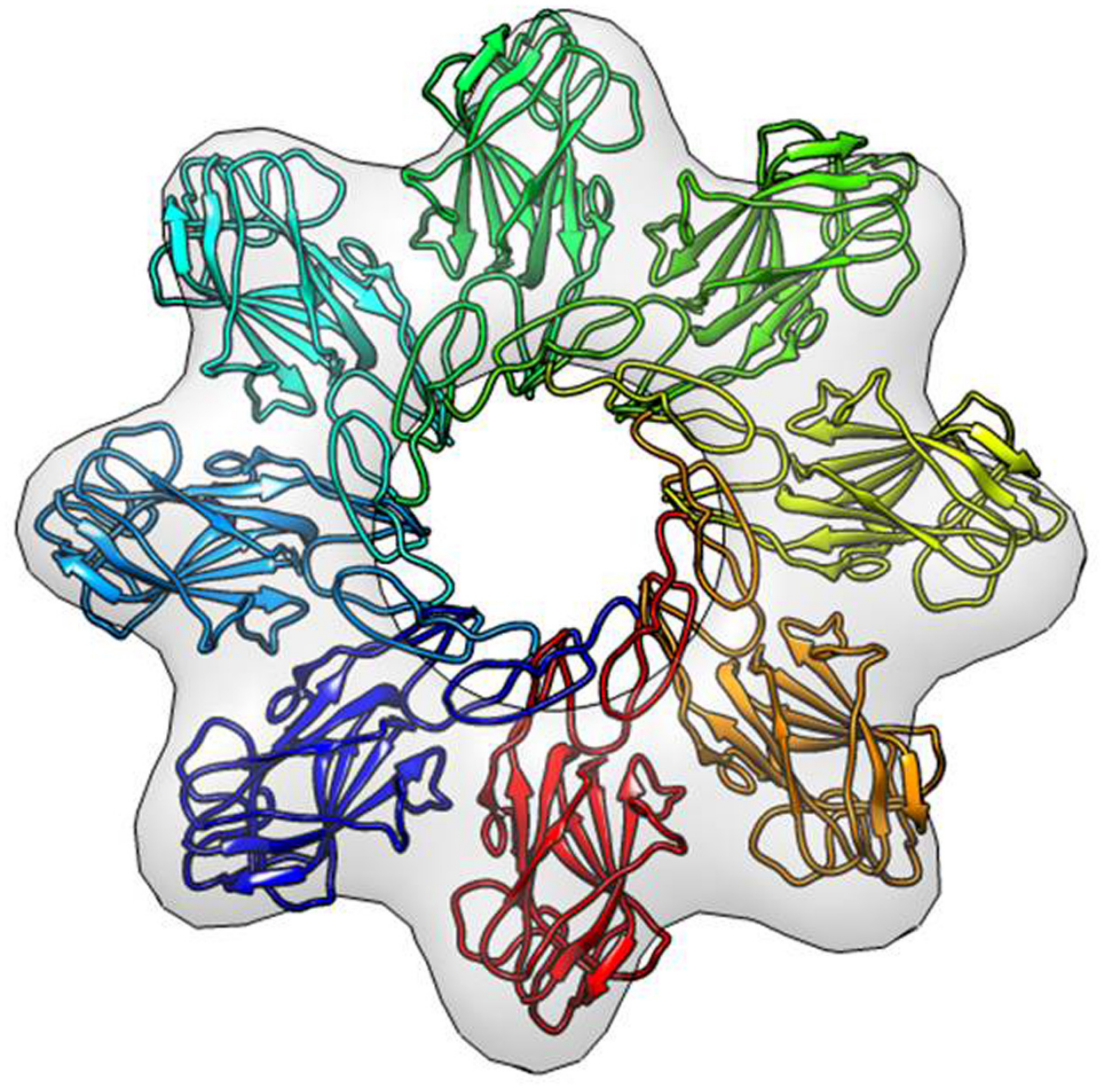
Modelling RS1 into EM map. Eight RS1 monomers fit within the EM framework. The discoidin domains project outward and the intermolecular disulphide bonds in the inner ring are essential for maintaining the octameric structure.

Using Chimera [25], we were able to sequentially fit the eight copies of the RS1 homology model into the EM density map and generate a pseudoatomic model of the RS1 octamer (Fig 5). In the modelled octamer, the monomers are oriented with the discoidin domain projecting outward from the central ring structure. The Rs1 domain is positioned within the inner ring enabling one subunit to contact an adjacent subunit and form an intermolecular disulphide bond. Previous biochemical studies suggest that disulphide bonds are the principal interaction between subunits since the octamer complex does not form when C59 and C223 are mutated to serine residues [11]. Interestingly, the hydrophobic spikes in the discoidin domain appear to fit in the space joining the two rings and could potentially serve to link the two rings as observed in the EM images.

## Discussion

In this study we have visualized for the first time the octameric RS1 complex by single particle EM and generated a molecular model of this oligomer that provides insights into its subunit organization. Notably, RS1 adopts a symmetrical cog-wheel structure with eight teeth projecting from the central ring with each tooth representing a subunit [11]. Molecular modelling studies support the view that the discoidin domains, the principal functional units of RS1, protrude from the central ring for binding to its cognate ligand and the ring structure is held together by intermolecular disulphide bonds. Double cog-wheel structures are observed in many EM images. The hydrophobic nature of the spikes in the discoidin domains appears to promote the stacking of two cog-wheels. The double cog-wheel structure would further increase the binding potential of RS1 to its cell surface ligand through multiple interactions. However, it is unclear if the double cog-wheel structure represents the native RS1 structure or an artefact of the negative staining procedure. Similar stacking has been observed in EM studies of negatively stained oligomeric proteins with ring-shaped structures [26, 27].

Although our studies provide new insight into the structural organization of the RS1 oligomeric complex, it will be important to more precisely define the structure of RS1 subunits using high resolution techniques. The relatively large size of the oligomeric complex may preclude the use of NMR methods. However, it should be possible to determine the structure by X-ray crystallography. We are continuing our studies directed toward obtaining suitable crystals for high resolution structural analysis. Alternatively, it should be possible to obtain near-atomic resolution (~3–4 Å) of the RS1 complex using single particle cryo-electron microscopy (cryo-EM). This technique has the advantage that it does not require large quantities of proteins or well-defined crystals for structural determination. Near resolution structures of a number of proteins and protein assemblies including the TRPV1 channel, ribosomes and viral particles have been determined using cryo-EM [28–30]. This technique could further delinate between a single and double cog-wheel structure of RS1 and more precisely define the RS1 subunit structure and oligomeric organization that is not possible by molecular modelling.

The precise physiological function of RS1 is not well-understood. The presence of cysts and cavities in the retina of XLRS patients and RS1 knockout mice has led to the suggestion that RS1 may function as a cell adhesion protein to glue the retina together. Alternatively, RS1 may regulate the fluid balance between the intracellular and extracellular environments of the retina. Several studies have shown that RS1 binds to the Na/K ATPase (α3,β2 isoform) on the surface of photoreceptors and bipolar cells [31, 32], most likely through the highly glycosylated β2-subunit. This interaction could modulate the activity of this ion pump thereby regulating retinal fluid balance. Optical coherence tomography has confirmed the presence of fluid-filled cystic cavities in the RS1 knockout mice [4]. Furthermore, large cavities in XLRS patients have been reduced in size with the treatment of carbonic anhydrase inhibitors [33, 34]. The creation of fluid-filled cavities could cause the disruption in cellular organization and synaptic structure and function of the retina as found in XLRS patients and RS1 knockout mice.

Phosphatidylserine (PS) has also been proposed to serve as a surface ligand for binding RS1 to retinal cell surfaces. In one study RS1 has been reported to bind to bilayers containing a high content of PS in the presence of Ca ions [35]. The C2 discoidin domain of Factor V is known to bind PS that is exposed on the surface of platelets during blood coagulation. However, under normal conditions PS is not typically found on cell surfaces and therefore it is unlikely that PS serves as a cell surface ligand for RS1 in the retina. Furthermore, other studies have indicated that RS1 does not associate with liposomes containing PS [31, 32].

L-type voltage-gated calcium channels in the retina have also been reported to bind RS1 in chicken retina [36]. This interaction has been implicated in photoreceptor-bipolar synaptic transmission and circadian rhythm. If confirmed, the reduction in the b-wave of ERGs in RS1 deficient mice and XLRS patients could be explained by the loss in regulation of voltage-gated calcium channels by RS1. It also would suggest that RS1 may interact with multiple cell surface receptors in the retina.

Although the mechanism by which RS1 maintains the integrity of the retina remains to be determined, genetic and biochemical studies indicate that the core homo-octameric structure is essential for its function as an extracellular protein. RS1 with either the C59S or C223R XLRS disease-causing mutation is expressed and secreted from cells, but fails to form the disulphide-linked octamer [10]. Such mutants also fail to bind to galactose affinity columns [14]. The multivalent nature of the single or double cog wheel complex most likely confers increased affinity for the binding of RS1 to its cell surface ligand analogous to the multiple interactions of a sprocket with a chain on a bicycle. In contrast to the C59S and C223R mutations, disease-causing missense mutations in the discoidin domains cause protein misfolding and aggregation together with retention of RS1 in endoplasmic reticulum by the quality control system of the cell [10, 37].

## Supporting Information

S1 Fig Refined images of the 50 classes of RS1 observed in 2-dimensions. (PDF)
