# Supplementary figures and images for: Cog-Wheel Octameric Structure of RS1, the Discoidin Domain Containing Retinal Protein Associated with X-Linked Retinoschisis
Source: PLoS One. 2016 Jan 26;11(1):e0147653. doi: 10.1371/journal.pone.0147653 (PMC4728063; doi:10.1371/journal.pone.0147653)

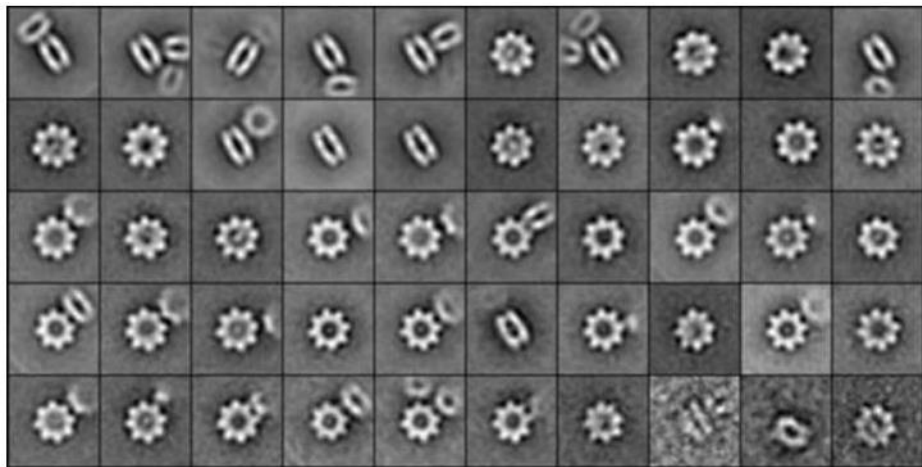

**S1 Fig.** Refined images of the 50 classes of RS1 observed in 2-dimensions.

Supplement: S1 Fig — (PDF) [file pone.0147653.s001.pdf]
